# Spontaneous Colitis in Muc2-Deficient Mice Reflects Clinical and Cellular Features of Active Ulcerative Colitis

**DOI:** 10.1371/journal.pone.0100217

**Published:** 2014-06-19

**Authors:** Ulf A. Wenzel, Maria K. Magnusson, Anna Rydström, Caroline Jonstrand, Julia Hengst, Malin EV. Johansson, Anna Velcich, Lena Öhman, Hans Strid, Henrik Sjövall, Gunnar C. Hansson, Mary Jo Wick

**Affiliations:** 1 Department of Microbiology and Immunology and the Mucosal Immunobiology and Vaccine Center (MIVAC), Institute of Biomedicine at Sahlgrenska Academy, University of Gothenburg, Gothenburg, Sweden; 2 Department of Internal Medicine and Clinical Nutrition, Institute of Medicine at Sahlgrenska Academy, University of Gothenburg, Gothenburg, Sweden; and MIVAC, University of Gothenburg, Gothenburg, Sweden; 3 Department of Medical Biochemistry, Institute of Biomedicine at Sahlgrenska Academy, University of Gothenburg, Gothenburg, Sweden; and MIVAC, University of Gothenburg, Gothenburg, Sweden; 4 Department of Oncology, Albert Einstein Cancer Center/Montefiore Medical Center, New York City, New York, United States of America; Charité, Campus Benjamin Franklin, Germany

## Abstract

**Background:**

The colonic mucus layer plays a critical role in intestinal homeostasis by limiting contact between luminal bacteria and the mucosal immune system. A defective mucus barrier in animal models allows bacterial contact with the intestinal epithelium and results in spontaneous colitis. A defective mucus barrier is also a key feature of active ulcerative colitis (UC). Alterations in the immune compartment due to intestinal bacterial breach in mice lacking the colon mucus barrier have not been characterized and correlated to active UC.

**Aims:**

To characterize alterations in the immune compartment due to intestinal bacterial breach in Muc2^−/−^ mice, which lack the colon mucus barrier, and correlate the findings to active UC.

**Methods:**

Bacterial contact with colon epithelium and penetration into colon tissue was examined in Muc2^−/−^ mice and colon biopsies from patients with active UC using fluorescence microscopy and qPCR. Neutrophils, lymphocytes, CD103^+^ dendritic cell subsets and macrophages in colon from Muc2^−/−^ mice and biopsies from UC patients were quantitated by flow cytometry.

**Results:**

Inflamed UC patients and Muc2^−/−^ mice had bacteria in contact with the colon epithelium. Bacterial rRNA was present in colonic mucosa in humans and Muc2^−/−^ mice and in the draining lymph nodes of mice. Inflamed Muc2^−/−^ mice and UC patients had elevated colon neutrophils, T cells and macrophages while a reduced frequency of CD103^+^ DCs was present in the inflamed colon of both mice and humans.

**Conclusions:**

The parallel features of the colon immune cell compartment in Muc2^−/−^ mice and UC patients supports the usefulness of this model to understand the early phase of spontaneous colitis and will provide insight into novel strategies to treat UC.

## Introduction

The colon lumen contains a massive amount of commensal bacteria that make essential contributions to metabolism and homeostasis, but aberrant reactivity to commensals can result in inflammatory bowel disease in genetically susceptible individuals. Indeed, the colonic barrier plays an important role to ensure that there is normally minimal contact between luminal bacteria and the mucosal immune system [1], [2]. The thick mucus layer covering the epithelium is a critical component of the colonic barrier by providing a physical as well as a chemical barrier harboring antimicrobial peptides [3]. The mucus barrier consists of a superficial loose outer layer a deep, adherent inner layer that is devoid of bacteria [1], [4]. In addition to the mucus layer, the epithelial layer is sealed by tight junctions between the cells that contribute to barrier function.

Ulcerative colitis (UC) is an idiopathic inflammatory bowel disease characterized by mucosal inflammation restricted to the colon that is accompanied by recurrent periods of bloody, loose stools [5]. It almost invariably affects the rectum and may involve the sigmoid colon or the entire colon. UC patients have a substantial risk for malignant transformation of the affected mucosa necessitating regular colonoscopy follow-ups to screen for colon cancer.

Despite massive research efforts, the etiology of UC remains elusive. Research has largely focused on the mucosal immune system, with the hypothesis that an imbalance in pro- and anti-inflammatory cytokines generates a vicious cycle leading to massive mucosal immune activation. UC patients indeed have alterations in many aspects of immune homeostasis that promote inflammation and propagate the disease [6], [7] but the initiating factors are poorly understood. However, evidence supports that inappropriate reactivity to luminal bacteria in genetically susceptible hosts plays an important role in disease pathogenesis [5]. Consistent with this, a defective colon barrier function is a feature of UC that allows increased bacterial contact with the intestinal epithelium that triggers immune activation [8], [9]. Experimental evidence supporting a defective barrier function in UC is reflected by the occurrence of spontaneous colitis in several mouse models where key components of barrier function are defective. The most straightforward model for a defective mucus barrier is Muc2^−/−^ mice, where Muc2, the mucin responsible for generating the mucus barrier in the colon, has been knocked out [10]. Muc2^−/−^ mice develop spontaneous colitis and have luminal bacteria in contact with the intestinal epithelium [1], [8]. Similarly, IL10-knockout mice, which also develop spontaneous colitis, have a defective mucus barrier with increased permeability to fluorescent beads the size of bacteria [8], [11]. Interestingly, increased mucus penetrability to beads was also seen in humans with active UC, and in a subgroup of patients with UC in remission [8].

Deciphering the etiology of UC as a means to developing new treatments would benefit from studies in pre-clinical models that, for example, allow the role of immune cells at early onset of colitis to be elucidated. Several pre-clinical models for UC have been developed where colitis is induced with chemicals like DSS or where effector T cells have been completely ablated [12], [13]. Induced models, however, have the disadvantage that the mechanism of how colitis is triggered is not understood. Therefore, provoked responses not mirroring the situation in patients are certainly possible, which limits the usefulness of induced models. Alternative models where colitis arises spontaneously in the absence of cell ablation or chemical induction would be a great advantage.

Here we take advantage of the spontaneous colitis that arises in Muc2^−/−^ mice to investigate the immune events that occur in inflammation. These events are correlated to findings in UC patients with active inflammation. Moreover, the inflammatory features of Muc2^−/−^ mice and inflamed UC patients are analyzed in relation to intestinal dendritic cell (DC) subsets defined by CD103 expression that may contribute to the early onset of colitis. Given the pivotal role of DCs in inducing T cell responses, characterizing changes in colon DC subsets that accompany the onset of spontaneous colitis will provide valuable insight into novel strategies to treat the chronic inflammation that plagues UC patients.

## Materials and Methods

### Mice

**Figure 1:**
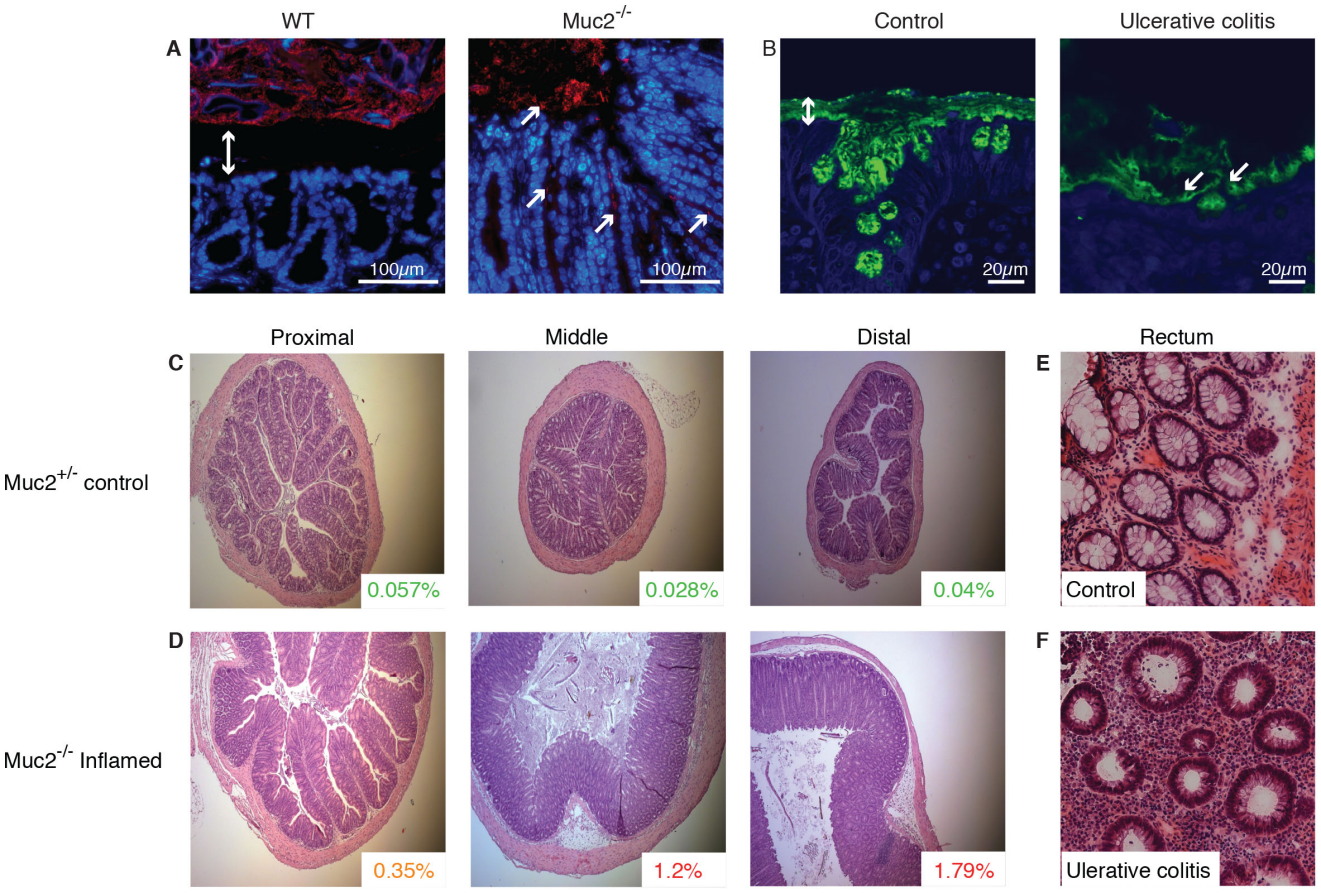
Histology of colon tissue from Muc2^−/−^ mice and UC patients. (**A**) Carnoy fixed whole colon sections with preserved mucus from WT and Muc2^−/−^ mice were analyzed by FISH (red) for bacteria localization and counterstained with DAPI. The mucus separating bacteria from the epithelium in WT mice is indicated by a double arrow and bacteria in contact with the epithelium in Muc2^−/−^ mice are marked by arrows. Scale bars are 100 µm. (**B**) Sigmoid sections of human biopsies from a control patient and a patient with active UC (Mayo endoscopic score 2) were stained for MUC2 (green) and DAPI. Mucus separating bacteria and the epithelium is indicated by a double arrow and bacteria within the remaining mucus in the UC patient are marked by arrows. Scale bars are 20 µm. (**C–D**) Representative sections from the proximal, middle or distal colon from (**C**) Muc2^+/−^ (18 weeks of age) and (**D**) inflamed Muc2^−/−^ (18 weeks of age) are shown. The neutrophil frequency determined by flow cytometry in parallel samples of the displayed tissues is indicated in the inlays. Original magnification is 5x. (**E–F**) Human rectal tissue from representative sections of (**E**) an inflamed UC patient at the time of diagnosis and (**F**) a non-inflamed control are shown. Original magnification is 40x. All tissues were sliced in 5 µm sections and stained with H&E.

Muc2^−/−^ mice backcrossed on C57BL/6 background between 8–19 weeks of age were used. These mice develop spontaneous colitis between 8 to 20 weeks of age, which often remains asymptomatic. This differs from Muc2^−/−^ mice kept on a 129Sv background that show features of colitis already at weaning [14]. Muc2^−/−^ mice on the C57BL/6 background were bred as Muc2^−/−^ × Muc2^+/−^ at the Experimental Biomedicine animal facility at the University of Gothenburg and offspring were genotyped [10]. C57BL/6 mice (Taconic, Denmark) were used as controls in fluorescence microscopy (Fig. 1A) while C57BL/6 (Charles River, Germany) were initially used as controls in flow cytometry experiments and were replaced with littermate Muc2^+/−^ controls for the remainder of the study. C57BL/6 mice were used at 8–19 weeks of age except for two 7 week old mice. Mice were kept under specific pathogen-free conditions and were provided with food and water ad libitum. Protocols were approved by the government animal ethics committee and institutional animal use and care guidelines were followed (permits 310/2010 and 280–2012). The general health of mice was monitored several times per week for signs of inflammation and animals were weighed once per week. An experiment was performed when one or more mice in a cage showed any sign of inflammation such as rectal swelling, rectal bleeding, soft stool or no weight gain. Mice were sacrificed by cervical dislocation after isoflurane anaesthesia using an airstream of 2 l/min and 3.5% isoflurane.

### Human Samples

UC patients with active inflammation or in clinical remission as well as non-inflamed controls (Table 1) were recruited at the endoscopy unit, Sahlgrenska University Hospital, Gothenburg, Sweden and were included after informed, written consent. Tissue biopsies were acquired at routine colonoscopy and mucosal inflammation was assessed macroscopically by the examining gastroenterologist and microscopically by a pathologist. Biopsies from macroscopically inflamed areas of the colon of UC patients as well as biopsies from the colon of non-inflamed controls undergoing colonoscopy for other indications (i.e. polyps, weight loss) were used. For FACS analysis, non-inflamed controls and patients with previously diagnosed UC were recruited whereas for qPCR and histology, non-inflamed controls and UC patients at the time of diagnosis were included. The protocol was approved by the Regional Ethical Review Board in Gothenburg (permit 040-08).

**Table 1:** Patient demographics.

	Healthy controls	UC established disease	UC at initial diagnosis	UC in remission
**Number**	16	10	10	12
**Age (y)**				
** Median (range)**	57 (32–75)	42 (21–63)	31 (19–62)	48 (26–63)
**Gender ratio (male/female)**	9/7	7/3	8/2	8/4
**Duration of disease (y)**				
** Median (range)**	NA*	4 (1–20)	0	23 (10–48)
**Active inflammation**	0	10	10	0
**Medications**				
** Thiopurines**	0	1	0	0
** 5-ASA**	0	4	0	7
** Corticosteroids**	0	1	0	0
** Corticosteroids, 5-ASA**	0	2	0	0
** No treatment**	16	2	10	5

*NA; not applicable.

### Histology

Tissue samples for histology from mice were acquired before removal of fecal pellets and longitudinal dissection of the colon. The colon was divided into three equal-sized segments, and a 5 mm long piece was taken from the middle of each of these three segments and put in 4% formaldehyde for fixation. The tissue samples were paraffin embedded, sliced into 5 µm sections and stained with hematoxylin and eosin (H&E). Human tissue samples were embedded in Tissue Tek OCT media (Sakure Finetek), sliced into 5 µm sections using a cryostat and stained with H&E. Segments were examined in a blind fashion using a Zeiss Axioscop 2 mot plus microscope with an AxioCam MRc (Carl Zeiss) and analyzed using Axiovision, Release 4.8.1 software (Carl Zeiss).

### Fluorescence Microscopy

Distal colon with fecal material from C57BL/6 (WT mice) and Muc2^−/−^ mice was dissected and fixed in water-free Carnoy’s fixative (60% dry methanol, 30% chloroform, 10% glacial acetic acid). Paraffin embedded sections were dewaxed and hybridized with 10 ng/µl of a general bacterial 16SrRNA probe (EUB 338) conjugated to Alexa 555 and DNA was stained with DAPI [1]. Human biopsies from control and active UC were taken during colonoscopy. The Carnoy fixed and paraffin embedded sections were immunostained for Muc2 using the MUC2C3 antisera and DAPI to visualize the nucleus and bacteria as not all mucus-associated bacteria in human samples can be stained by the general EUB338 probe [8]. Images were obtained using a fluorescence microscope Eclipse E1000 with a Plan-Fluor 40x/0.75 DIC objective (Nikon) or a confocal microscope Axio Examiner Z1 LSM 700 with a plan apochromat 40×/1.3 oil DIC objective and the ZEN 2010 software (Zeiss, Germany).

### Preparation of Mouse and Human Tissues

Mice were sacrificed at 8–19 weeks of age. The colon was loosened from fat and luminal contents were removed. The colon was opened longitudinally and rinsed with PBS. Tissue was cut into small pieces that were kept on ice in calcium and magnesium-free (CMF) HBSS containing 1.5 mM Hepes. The supernatant was discarded and tissue pieces were treated with HBSS-EDTA (CMF HBSS supplemented with 2% FCS (PAA; Pasching, Austria), 1.5 mM Hepes (Gibco Life Technologies, Auckland, New Zealand) and 2 mM EDTA) for 15 minutes at 37°C. Samples were then vigorously shaken and filtered using a nylon mesh. The procedure was repeated for a total of three EDTA treatments followed by a wash in RPMI 1640 medium supplemented with 10% FCS and 1.5 mM Hepes. Single cell suspensions were prepared by a 45 minute incubation at 37°C with 100 U/ml collagenase type VIII (Sigma-Aldrich) and 60 U/ml DNase I (Sigma-Aldrich) diluted in RPMI 1640 supplemented with 10% FCS, 1.5 mM Hepes and 2 mM CaCl_2_. Following digestion, supernatants were collected by filtration through a nylon mesh and viable cells were counted using trypan blue exclusion. Colon biopsies from patients were collected in PBS, immediately put on ice and cells were isolated as described for mouse tissue.

### Flow Cytometry

Single cell suspensions of mouse lamina propria (LP) cells were stained in HBSS containing 3% FCS, 5 mM EDTA and 20 mM HEPES. Samples were first treated with anti-FcγR III/II monoclonal antibody (clone 2.4G2) for 15 min at 4°C. Cells were washed, antibody cocktails were added and the cells were incubated for 20 min at 4°C. 7-Aminoactinomycin D (7AAD, Sigma–Aldrich) or Live/Dead Fixable Aqua Dead Cell Stain Kit (Gibco Life Technologies, Auckland, New Zealand) were used to exclude non-viable cells according to the manufacture’s protocol. The following monoclonal antibodies recognizing mouse molecules conjugated to the indicated fluorochromes were used: anti- MHC-II-Alexa700, TCR-β-APC, CD19-APC, NK1.1-APC, CD11b-APC-Cy7, Ly6G-FITC, CD11c-Pacific Blue, CD103-PE, B220-PE-Cy7, CD4-APC and CD8-Pacific blue. Mouse antibodies were from BD Biosciences except MHC-II Alexa fluor 700 (eBioscience, San Diego, CA).

Following surface staining, intracellular iNOS staining was performed by fixing the samples in 2% formaldehyde in PBS for 20 minutes at room temperature followed by a wash in permeabilization buffer (HBSS containing 0.5% saponin and 0.5% BSA (Sigma-Aldrich)) and a subsequent incubation for 30 minutes at room temperature with either anti-iNOS Ab (M-19) (Santa Cruz Biotechnology) or appropriate isotype control in permeabilization buffer. The incubation was followed by an additional incubation with allophycocyanin-conjugated anti-rabbit IgG (Santa Cruz Biotechnology) for 30 min at room temperature.

Single cell suspensions of human biopsies were stained by excluding non-viable cells using 7AAD and adding anti- CD3-FITC+CD19-FITC as an exclusion cocktail as well as anti- CD14-PeCy7, CD103-APC, CD11c-Pacific blue and HLA-DR-Alexa700. All human antibodies were from BD Biosciences except CD103-APC (eBioscience) and HLA-DR-Alexa700 (BioLegend, San Diego, CA). Both human and mouse samples were collected with a LSRII flow cytometer (BD Biosciences) using DIVA software (BD Biosciences) and analyzed using FlowJo software (Tree Star, Ashland, OR).

### Quantitative Real Time PCR (qPCR)

Tissues and cells were lysed using QIAGEN tissue-lyser according to the manufacture’s protocol. Total RNA was extracted with the High Pure RNA Tissue Kit (Roche Diagnostics) according to the manufacturer’s protocol. RNA quantity and purity were measured using a NanoDrop ND-1000 Spectrophotometer (NanoDrop Technologies, Inc.). RNA was reverse transcribed with the Transcriptor First Strand cDNA Synthesis Kit (Roche Diagnostics) and qPCR was performed on a LightCycler480 thermal cycler (Roche) using LightCycler480 SYBR Green Master (Roche). Primers for 18srRNA [15], 23srRNA (fwd-5′ GAA GGA ACT AGG CAA AAT GG 3′; rws-5′ GGG TAC ACT GCA TCT TCA CA 3′), HPRT (fwd-5′ TCC TCC TCA GAC CGC TTT T 3′; rws-5′ CCT GGT TCA TCA TCG CTA ATC 3′) CCL2 (fwd-5′ GAT CAT CTT GCT GGT GAA TGA GT 3′; rws-5′ CAT CCA CGT GTT GGC TCA 3′), CXCL2 (fwd-5′ CTT TGG TTC TTC CGT TGA GG 3′; rws-5′ AAA ATC ATC CAA AAG ATA CTG AAC AA), IFN-γ (fwd-5′ TTC AAG ACT TCA AAG AGT CTG AGG TA 3′; rws-5′ ATC TGG AGG AAC TGG CAA AA 3′), IL-6 (fwd-5′ CCA GGT AGC TAT GGT ACT CCA GAA 3′, rws-5′ GCT ACC AAA CTG GAT ATA TCA GGA 3′), IL-10 (fwd-5′ GTC CAG CTG GTC CTT TGT TT 3′; rws-5′ CAG AGC CAC ATG CTC CTA GA 3′), IL-17a (fwd-5′ GCT GAG CTT TGA GGG ATG AT 3′; rws-5′ CAG GGA GAG CTT CAT CTG TGT 3′) iNOS (fwd-5′ CCA TGA TGG TCA CAT TCT GC 3′; rws-5′ GGG CTG TCA CGG AGA TCA 3′) TNF-α (fwd-5′ TGC CTA TGT CTC AGC CTC 3′; rws-5′ GAG GCC ATT TGG GAA CTT CT 3′), TGF-β (fwd-5′ TGG AGC AAC ATG TGG AAC TC 3′; rws-5′ CAG CAG CCG GTT ACC AAG 3′and Relmβ (fwd-5′ GCA CAT CCA GTG ACA ACC AT 3′; rws-5′ GGA AGC TCT CAG TCG TCA AGA 3′) were designed using Primer3 software and purchased from Eurofins MWG Operon (Ebersberg, Germany). Specificity and efficiency was tested in initial analyses. Bacterial burden was assessed using the 2^ΔΔCt^-Method [16] normalizing to the Ct-value of 18srRNA. Differential gene expression of Relmβ was determined using the 2^ΔΔCt^-Method normalizing to the Ct-value of HPRT.

### Statistical Analysis

Statistical analyses were performed with GraphPad Prism 5.0 (GraphPad Software, La Jolla, CA). Wilcoxon signed rank test was used to evaluate differences between two paired groups. For comparison of two independent groups, the two-tailed nonparametric Mann-Whitney-U test was applied while Kruskal-Wallis test followed by Dunn’s multiple comparison was used for comparison between three or more groups. Pearson correlation was performed to check for correlation between parameters. A p value below 0.05 was considered statistically significant.

## Results

### Compromised Mucus Barrier and Aberrant Histological Features in the Colon of Muc2^−/−^ Mice and UC Patients

To evaluate Muc2-deficient mice as a model for UC, with focus on the early phase of the disease, Muc2^−/−^ and Muc2^+/−^ mice were monitored from age 8 weeks onward for one or more of the following visual signs of colitis: rectal swelling, rectal bleeding, soft stool or no weight gain. When some mice in a cage exhibited one of these signs, with rectal swelling being the most frequent, these apparently colitic mice, as well as Muc2^−/−^ littermates without visual signs of inflammation and Muc2^+/−^ controls, were sacrificed for investigation.

We first examined the integrity of the mucus barrier, which normally separates luminal bacteria from the epithelium in the distal colon [1], [2]. Mucus clearly separates bacteria from the epithelial layer in WT mice whereas bacteria were observed on the epithelium and in crypts of Muc2^−/−^ mice [1], [8] (Fig. 1A). This occurs in all Muc2^−/−^ mice as a result of a non-existing mucus layer and may differ from humans where a known genetic defect has not completely removed the mucus layer. However, despite that the Muc2^−/−^ model is an extreme form of mucus layer defect, similarities between Muc2^−/−^ mice and UC patients are apparent. For example, in colon biopsies from humans, non-inflamed controls had a stratified mucus layer that separated bacteria from the epithelium while biopsies from UC patients with active colitis revealed bacteria within the mucus close to the epithelium (Fig. 1B). This is observed in the few biopsies that have a remaining mucus layer, as mucus is often lost by the mechanical manipulation during collection.

The defective mucosal barrier in Muc2^−/−^ mice and UC patients was accompanied by histological changes indicative of inflammation. For example, relative to Muc2^+/−^ controls, colitic Muc2^−/−^ mice had a distorted and flattened structure of the epithelial cells, loss of LP architecture, cell infiltration and superficial epithelial erosions that were more pronounced toward the distal end of the colon (Fig. 1 C–D). Moreover, colitis coincided with an increased crypt length, particularly in the distal colon (Fig. 1 C–D). The LP of Muc2^−/−^ had a significant increase in total cells, CD4^+^ and CD8^+^ T lymphocytes and IgA-producing B cells compared to WT mice (Fig. S1A–D). Human rectal samples from inflamed UC patients also revealed distorted epithelial cells, loss of architecture and massive cell infiltration relative to controls (Fig. 1 E–F) including an increased fraction of T lymphocytes (Fig. S1E). Together these results suggest similar alterations of mucus barrier integrity allowing bacterial contact with epithelial cells, as well as aberrant intestinal architecture, in colitic Muc2^−/−^ mice and patients with active UC. Also, the inflammatory signs in Muc2^−/−^ mice increase towards the distal end of the colon, which is a key feature of UC [5].

### Luminal Bacteria Penetrance and Triggering of Anti-bacterial Effectors

**Figure 2:**
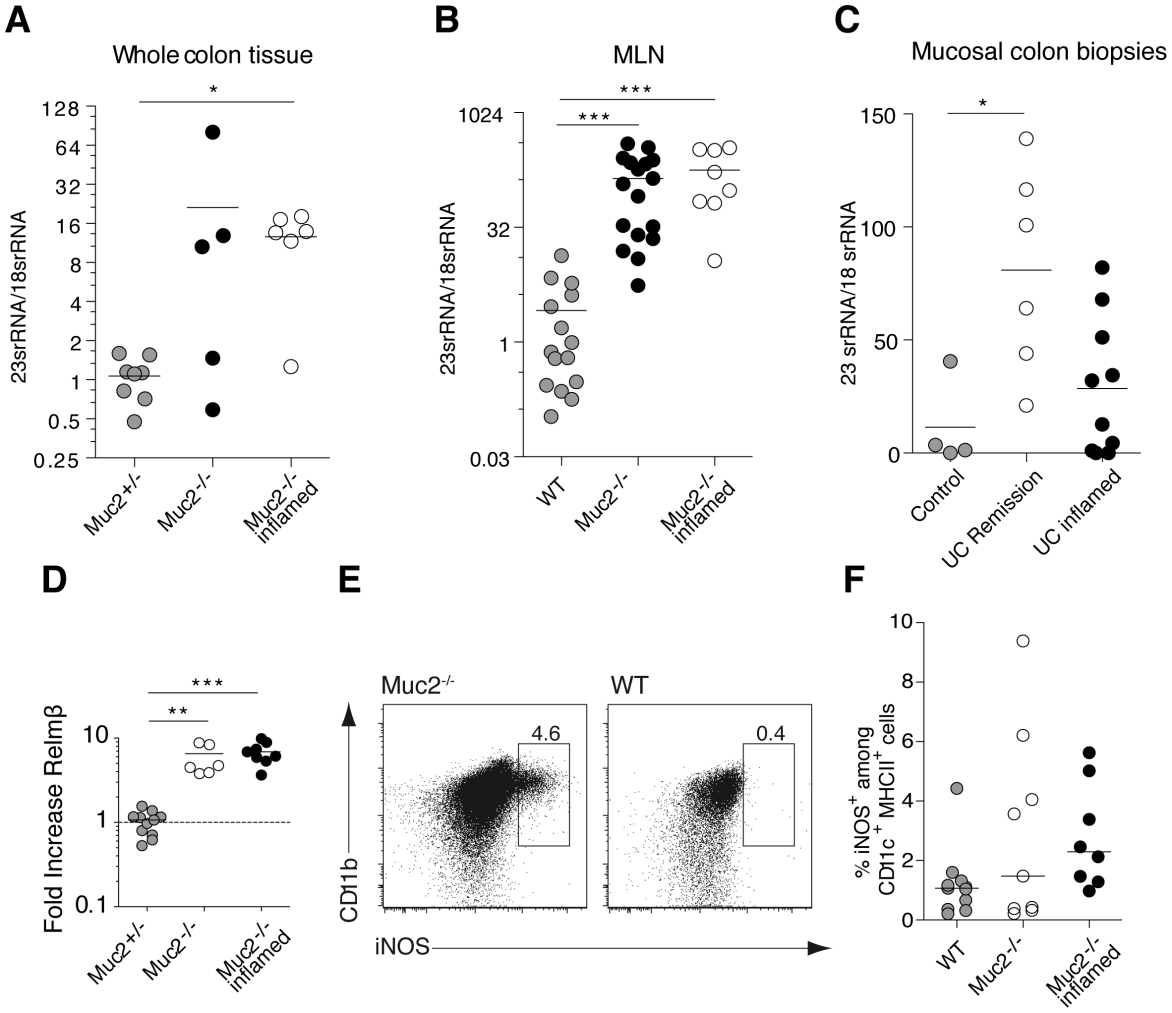
Luminal bacteria penetrate colon tissue of Muc2^−/−^ mice and inflamed UC patients. qPCR was used to determine the ratio of 23SrRNA to 18SrRNA to assess bacterial presence in mouse colon tissue (**A**), mouse MLN (**B**) and biopsies from UC patients with active inflammation, from UC patients in clinical remission and from non-inflamed controls (**C**). Results were analyzed using the ΔΔCT method with the CT value of 18SrRNA as the endogenous reference gene. (**D**) Differential gene expression of Relmβ was analyzed using the ΔΔCT method with the CT value of HPRT as the endogenous reference gene. (**E**) Dot plots show intracellular iNOS expression by gated viable (7AAD^−^) CD11c^+^MHC-II^hi^CD11b^+^ cells from the MLN of a representative inflamed Muc2^−/−^ mouse (left) and a WT mouse (right). (**F**) Scatter plot show the percent iNOS-expressing cells among total CD11c^+^MHC-II^hi^CD11b^+^ cells from the MLN of WT mice (grey circles), Muc2^−/−^ mice (open circles) and inflamed Muc2^−/−^ mice (black circles). Mice in the “Muc2^−/−^ inflamed” group had a higher frequency of colon PMNs relative to other Muc2^−/−^ mice indicating they were colitic and were analyzed as a separate group; see text for details. Mouse data was obtained from 3 independent experiments with 8–11 mice in each group. Each symbol represents an individual. Mice were between 8–16 weeks of age except 2 WT mice that were 7 weeks old. Statistical significance between groups was assessed using the Kruskal-Wallis test followed by Dunn’s multiple comparison test (*p<0.05, **p<0.01, ***p<0.001).

Defects in the inner mucus barrier, which normally separate luminal bacteria from intestinal epithelial cells, may trigger colitis in both Muc2^−/−^ mice [1] and UC patients [6]–[8]. We thus extended the microscopy data in Figure 1A–B and previous in situ studies [1], [8] by using quantitative RT-PCR (qPCR) to determine luminal bacterial penetration into the colon and draining lymph nodes. Consistent with a defective mucus barrier (Fig. 1A), Muc2^−/−^ mice had higher bacterial burden than control mice in colon tissue and mesenteric lymph nodes (MLN) (Fig. 2A–B). This occurred in Muc2^−/−^ mice irrespective of ongoing inflammation (assessed by PMN influx; see below). 23SrRNA was also quantified as a means to assess bacterial load in human rectal samples from inflamed UC patients at the time of initial diagnosis, before the influence of any anti-inflammatory treatment, and in UC patients in remission. Data from these patients also showed a trend towards an increased bacterial load compared to controls (Fig. 2C). The qPCR data in Fig. 2C supports the heterogeneity in mucus quality defects in UC patients with Mayo score 0–3 demonstrated using a bead penetrability assay [8].

To further demonstrate bacterial influence on epithelial cells of Muc2^−/−^ mice, expression of the colon-restricted anti-microbial effector Relmβ, whose expression is rapidly induced upon conventionalization of germ-free mice [17], was quantitated. Relmβ was up-regulated in colon epithelial cells of Muc2^−/−^ mice regardless of their inflammatory status assessed by PMN influx (Fig. 2D). Detection of Relmβ mRNA in all Muc2^−/−^ mice supports bacterial contact with the intestinal epithelium of all Muc2^−/−^ mice while only a subset of Muc2^−/−^ mice showed signs of inflammation based on other indicators of inflammation such as PMN influx at the time of analysis. Indeed, production of the anti-microbial effector inducible nitric oxide synthase (iNOS), which is induced in myeloid cells upon sensing luminal bacterial infection [18], was also induced in myeloid cells in the MLN of some Muc2^−/−^ mice (Fig. 2E–F). Expression of proinflammatory cytokines and chemokines was not correlated to mouse age *per se* (Fig. S2B). Thus, the defective inner mucus layer that allows bacterial contact with intestinal epithelial cells (Fig. 1A and 2D) is accompanied by increased bacterial presence in colon tissue of Muc2^−/−^ mice and UC patients. Moreover, luminal bacteria are detected in the MLN of Muc2^−/−^ mice where the anti-microbial effector molecule iNOS is induced.

### PMN Influx in the Colon is an Early Feature of Inflammation

**Figure 3:**
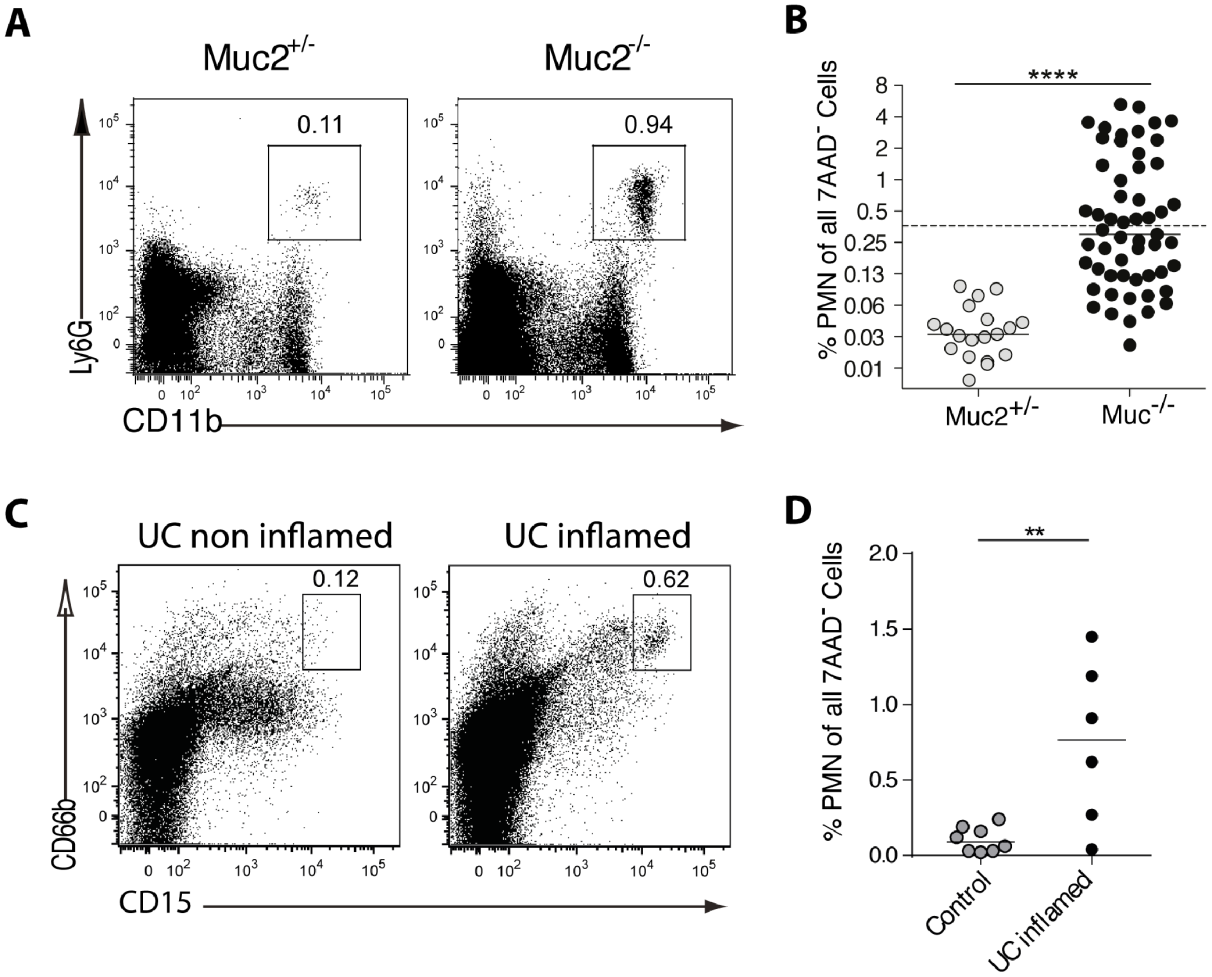
PMNs increase in the colon of Muc2^−/−^ mice and UC patients. Colon LP cells from Muc2^−/−^ mice, Muc2^+/−^ littermates, UC patients and non-inflamed controls were analyzed by flow cytometry. (**A**) PMNs in mice were identified as viable (7AAD^−^) MHCII^−^CD11b^+^Ly6G^+^ cells. The numbers represent the percent cells in the indicated gate. The Muc2^−/−^ mouse shown did not have overt signs of inflammation such as rectal swelling. (**B**) The frequency of PMNs among viable LP cells for all mice examined is shown. The dashed line indicates the “cut off” value of 0.36% (see text for details). Results are from 17 independent experiments with a total of 19–55 mice per group. (**C**) PMNs in human tissue were identified as 7AAD^−^MHCII^−^CD15^hi^CD66b^+^ cells. The numbers represent the percent cells in the indicated gate. PMN gating was performed using a non-inflamed and inflamed sample from different colon regions from the same UC patient as negative and positive control. (**D**) The frequency of PMNs among viable LP cells for non-inflamed controls and UC patients with established disease is shown. Statistical significance was assessed using the Mann-Whitney-U-Test; significance is indicated as **p<0.01, ****p<0.0001. Each symbol represents an individual mouse or patient. The mice used were between 8–19 weeks of age.

The defective mucosal barrier and luminal bacteria penetration in Muc2^−/−^ mice, which is paralleled in UC patients [8], [19] (Figs. 1A and 2), provides a unique opportunity to use Muc2^−/−^ mice to decipher early changes in the immune compartment that potentially drive colitis. PMNs are rare in quiescent tissue and rapidly increase when bacterial penetration is sensed [18] and are an early cellular indicator of inflammation. We thus quantitated colonic PMNs in a large number of Muc2^−/−^ mice as a means to identify animals with and without cellular indications of inflammation. Muc2^−/−^ mice displayed a broad range of PMN frequencies (0.05% to 5.97%; Fig. 3 A–B) that was not correlated to age (Fig. S2A). The high frequency of colon PMNs in some Muc2^−/−^ mice suggested that these animals had intestinal inflammation, even in the absence of clinical features. We used this observation to place Muc2^−/−^ mice into two working groups to correlate inflammation, defined as increased PMNs in colon LP, with other immune parameters. Specifically, a cut off value was set at 0.36% PMNs, which is twice the highest frequency of colon PMNs observed in any control animal (0.18%). Muc2^−/−^ mice with >0.36% colon PMNs were called inflamed and were compared with Muc2^−/−^ mice with <0.36% colon PMNs and Muc2^+/−^ controls. The dashed line in Figure 3B indicates this cut off value. PMN influx was not apparent in the distal small intestine of Muc2^−/−^ mice including those with PMN influx in the colon (Fig. S3A). The colon LP of UC patients also showed an increase of colon PMNs, identified as MHCII^−^CD15^+^CD66b^+^ cells (Fig. 3 C–D). Thus, elevated PMNs among colon LP cells are early indications of inflammation in Muc2^−/−^ mice and reflect inflammatory signs in UC patients. Elevated T cells in colon LP also characterized both Muc2^−/−^ mice and UC patients, while Muc2^−/−^ mice additionally had increased numbers of IgA-producing cells in colon LP (Fig. S1).

### Changes in Dendritic Cells and Macrophages Characterize Colon Inflammation in Muc2^−/−^ Mice

DCs and macrophages are important in coping with bacteria in tissues and present bacterial antigens to T cells, which in the case of bacterial penetration as seen in Muc2^−/−^ mice and UC patients, could drive colitis. This raises the possibility that the initiation of inflammation is mirrored in changes in colonic DCs or macrophages. To address this, we quantitated the frequency of colon DC subsets defined by expression of CD103 and CD11b. Functionally, CD103^+^CD11b^+^ DC carry antigen from the intestine and can induce Th17 cells while CD103^+^CD11b^−^ DC can induce Th1 or Th17 cells [20], [21]). We also quantitated colon macrophages [22], [23], identified as CD103^−^CD11b^+^ cells, in the colon LP of Muc2^−/−^ and Muc2^+/−^ mice.

**Figure 4:**
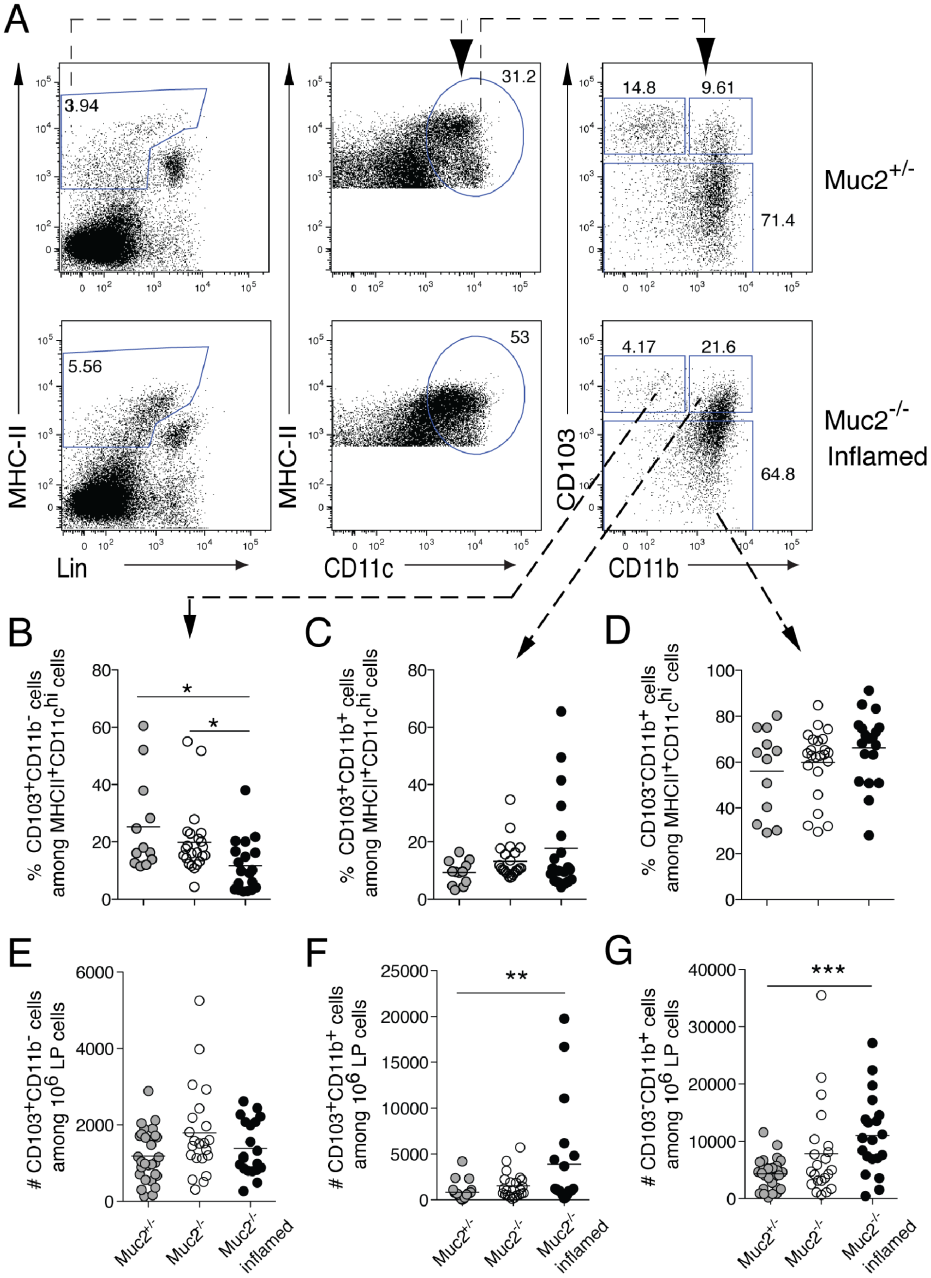
The composition of colon DCs and macrophages is altered in inflamed Muc2^−/−^ mice. LP cells from the colon of Muc2^−/−^ mice, inflamed Muc2^−/−^ mice and Muc2^+/−^ controls were stained for DCs and macrophages and analyzed by flow cytometry. (**A**) The gating strategy used is shown where viable MHC-II^+^NK1.1^−^TCR^−^CD19^−^ cells (left column) were distinguished by CD11c expression (middle) and CD11c^+^MHC-II^+^ cells were then analyzed for CD103 and CD11b (right). The numbers are the percent of cells in the indicated gates. (**B–D**) The frequency of CD103^+^CD11b^−^ DCs (**B**), CD103^+^CD11b^+^ DCs (**C**), and CD103^−^ macrophages (**D**) among MHCII^+^CD11c^hi^ cells in the indicated gates is shown. (**E–G**) The absolute number of CD103^+^CD11b^−^ (**E**), CD103^+^CD11b^+^ (**F**) and CD103^−^ macrophages (**G**) among 10^6^ viable LP cells is shown. Each symbol represents an individual mouse. The median is indicated by a horizontal line. Data are from 15 independent experiments that analyzed 20–29 mice per group. The mice were between 8–19 weeks of age. Statistical significance between groups was assessed using the Kruskal-Wallis test followed by Dunn’s multiple comparison test. Significance is indicated as *p<0.05, **p<0.01, while all other comparisons are non-significant.

The percent of CD103^+^CD11b^−^ DCs was reduced in the colon LP of inflamed mice, while the percent of CD103^+^CD11b^+^ DC and CD103^−^CD11b^+^ macrophages remained unchanged with inflammation (Fig. 4A–D). Analyzing the absolute number of the cells revealed that both CD103^+^CD11b^+^ DCs and CD103^−^CD11b^+^ macrophages increased in the colon LP of inflamed Muc2^−/−^ mice (Fig. 4E–G). Thus, the spontaneous inflammation characterized by neutrophil infiltration in the colon LP of Muc2^−/−^ mice corresponded with decreased frequency of CD103^+^CD11b^−^ DCs and increased number of CD103^+^CD11b^+^ DCs and CD103^−^CD11b^+^ macrophages in the colon. No alterations in the frequency or number of the DC or macrophage populations were apparent in the small intestine LP (Fig. S3B and data not shown).

### The Inflamed Colon of UC Patients is Characterized by Reduced CD103^+^ DCs and Increased CD14^+^ Macrophages

**Figure 5:**
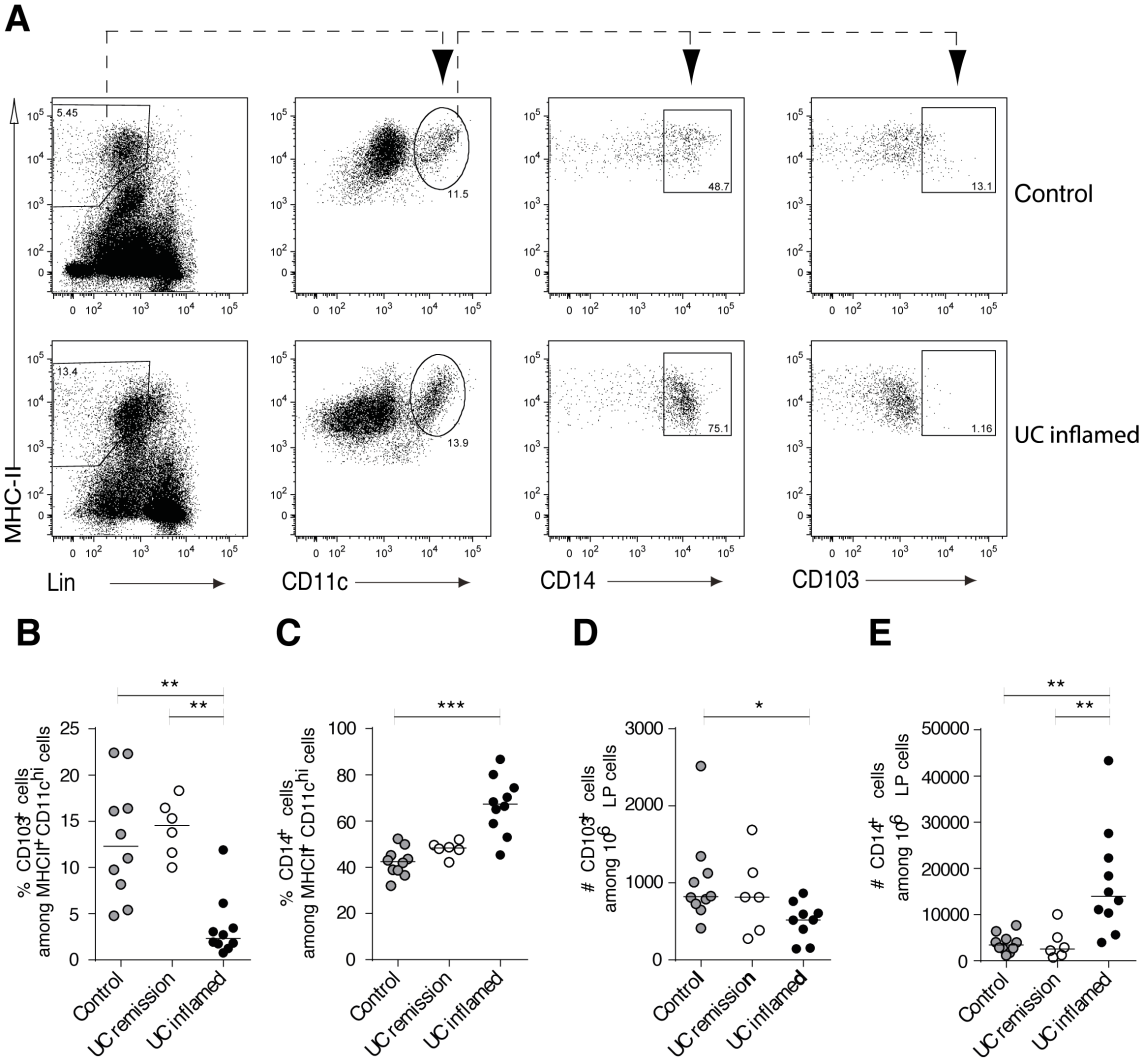
Altered abundance of DCs and macrophages characterizes the inflamed colon LP of UC patients. LP cells from biopsies taken from inflamed areas of UC patients with active inflammation, from UC patients in clinical remission and from non-inflamed controls were analyzed by flow cytometry. (**A**) The gating strategy used where viable CD3^−^CD19^−^HLADR^+^ cells that were CD11c^+^ (left two plots) were further analyzed to identify CD14^+^ macrophages and CD103^+^ DCs (right two plots). Data from a representative non-inflamed control and an inflamed UC patient are shown. (**B**) The percent of CD103^+^ DCs and (**C**) CD14^+^ macrophages among HLADR^+^CD11c^+^ cells is shown. The absolute number of (**D**) CD103^+^ DCs and (**E**) CD14^+^ macrophages among 10^6^ viable LP cells is shown. Each symbol represents an individual patient and the median is indicated by a horizontal line. Non-inflamed controls n = 10, UC patients in remission n = 6, UC patients with active inflammation n = 10. Statistical significance between groups was assessed using the Kruskal-Wallis test followed by Dunn’s multiple comparison test (*p<0.05, **p<0.01, ***p<0.001).

While mouse intestinal CD103^+^ DC have been extensively studied, little is known about human intestinal CD103^+^ DC in health versus disease. We thus analyzed changes in CD103^+^ DC, as well as CD14^+^ monocytes/macrophages, in inflamed colon tissue of UC patients to determine if alterations similar to those observed in Muc2^−/−^ were apparent. A significant decrease of CD103^+^ DC among HLADR^+^CD11c^+^ cells was apparent in inflamed colon tissue from UC patients relative to tissue from non-inflamed controls (Fig. 5A–B). In contrast, CD14^+^ monocytes/macrophages were increased in inflamed colon of patients relative to non-inflamed controls (Fig. 5C). Thus, similar to the Muc2^−/−^ model, a reduced fraction of colon lamina propria CD103^+^ DCs and increased CD14^+^ monocytes/macrophages correlates to inflammation in patients with active UC.

## Discussion

Here we use Muc2-deficient mice, which is an extreme form of a mucus layer defect resulting in luminal bacteria in contact with intestinal epithelial cells, as a spontaneous colitis model to investigate the early events contiguous with defective barrier-driven commensal reactivity that is reminiscent of UC [8], [9]. Indeed, a study of mucus quality in human colon showed mucus defects in inflamed UC patients as well as some uninflamed controls [8]. Although the mucus remaining in UC patients will be a bacterial hinder, this suggests that, similar to the severe defect in Muc2^−/−^ mice, mucus defects are a contributing factor for the development of UC in humans [8]. Similar to UC, inflammation in Muc2^−/−^ mice results in loss of colon architecture and increases proximally from the rectum. Importantly, signs of inflammation such as PMN influx and increased inflammatory cytokines were not apparent in the small intestine of Muc2^−/−^ mice. The colon-restricted nature of inflammation in Muc2^−/−^ mice mimics that in UC, where exclusive involvement of the colon is a hallmark and differs from Crohn’s disease, where the whole gastrointestinal tract can be involved. Moreover, some of the Muc2^−/−^ mice showed changes in fecal consistency and/or developed perianal swelling, features similar to UC disease progression in humans. We also show that colonic infiltration of PMNs and lymphocytes, as well as changes within the myeloid cell compartment, are present in the inflamed colon mucosa of UC patients and Muc2^−/−^ mice, strengthening the parallels of this model for UC. PMN influx seems to be an early indicator of inflammation even in the absence of clinical signs of colitis in both Muc2^−/−^ mice and patients [24], the latter being supported by measuring fecal calprotectin to monitor UC [25]. Further analysis of the two groups of Muc2^−/−^ mice that differ in colon PMN influx will provide valuable insight into the immune features and myeloid cell function in the transition to colitis. This can be correlated to UC patients at different stages of disease, such as newly diagnosed and actively inflamed, to better understand disease progression in humans and potentially develop new therapies.

The aberrant mucus barrier in Muc2^−/−^ mice enables commensal bacteria to penetrate not only into the colon LP but also to the MLN. Likely candidates for transporting bacteria from the intestine to the MLN, as well for subsequent initiation of a pathogenic T cell response, are intestinal migratory DCs, where the predominant migratory populations in mice express CD103 [26]. In inflamed human UC and Muc2^−/−^ intestinal samples we detected an overall increase in a broad antigen presenting cell population (CD11c^+^MHC-II^+^ cells). The altered composition of macrophages and DC subsets in colon LP during inflammation in Muc2^−/−^ mice are consistent with the observed increase in CD11c^+^ cells in mice with a missense mutation in Muc2 [27]. Moreover, spontaneous colitis in inflamed Muc2^−/−^ mice was accompanied by an increased absolute number of CD103^+^CD11b^+^ DCs and CD103^−^CD11b^+^ cells. This suggests that infiltration of these cell populations, rather than emigration or death of CD103^+^ DCs, underlies the altered balance of myeloid cell populations associated with colitis. The CD103^−^CD11b^+^ population that increased in colitis consists of F4/80^hi^ macrophages, Ly6C^hi^ monocytes and DCs [23], [26] (and data not shown). This is consistent with the accumulation of similar cell populations that drive local inflammation in induced colitis models such as T cell transfer or DSS [23], [28]–[34]. These induced models of colitis also showed a reduction in the fraction of CD103^+^ DC in colon LP. Thus, induced colitis models as well as spontaneous colitis in Muc2^−/−^ mice demonstrate that accumulation of CD103^−^CD11b^+^F4/80^int^ cells in LP with a concomitant reduction in the fraction of CD103^+^ DCs are hallmarks of colitis.

Importantly, we detect similar changes in UC patients. That is, local infiltration of CD14^+^ monocytes/macrophages accompanies PMN influx as well as reduced abundance of CD103^+^ DCs in the colon LP. It has been shown that CD14^+^ macrophages increase in the inflamed mucosa of Crohn’s and UC patients [22], [35]. Moreover, Kamada et al identified increased levels of CD14^+^CD33^+^ macrophages in the colon LP of Crohn’s disease and UC patients that play an important role in disease pathogenesis [36]. Similarly, Franzè et al showed accumulation of monocytes/macrophages identified by CD163 expression in inflamed biopsies of Crohn’s and UC patients [37]. Thus, the CD103^−^CD11b^+^ cells that increase in the LP of colitic Muc2^−/−^ mice may resemble the CD14^+^ monocytes/macrophages that increase in inflamed colon of UC patients. This provides the opportunity to dissect the function of these cells in driving colitis in a spontaneous colitis model. Little is known about DC subsets in intestinal mucosa of humans, particularly in inflammatory bowel disease. However, CD103^+^ DCs have been identified in healthy human colon, duodenum and MLN [38]–[40] and have been shown to decrease in celiac lesions [41]. Here, we also detected a reduction of CD103^+^ DCs in inflamed compared to non-inflamed colon. Given the importance of CD103^+^ DCs in intestinal homeostasis determined from murine studies [42], [43], deciphering the role of these cells in human IBD is clearly warranted.

Taken together, we have described a similar disturbance in myeloid cell populations in spontaneous colitis in Muc2^−/−^ mice and patients with active UC, which both have commensal barrier breach. Our data support that spontaneous colitis in Muc2^−/−^ mice is a valuable means to decipher the role of DC subsets and monocytes/macrophages in driving UC and provide valuable insight into new strategies to treat the inflammation that plagues UC patients.

## Supporting Information

Figure S1 **Lymphocytes increase in the colon LP of Muc2^−/−^ mice and UC patients with active inflammation.** LP cells were prepared from the colon of Muc2^−/−^ mice and WT mice and from biopsies from UC patients with active inflammation, from UC patients in clinical remission and non-inflamed controls and were analyzed by flow cytometry. **(A)** The median number of total, viable (7AAD−) cells from colon LP from 21 WT and 35 Muc2^−/−^ mice from 7 independent experiments is shown. **(B)** The percent B220^−^MHCII^−^CD8^−^CD4^+^ T cells or B220^−^MHC-II^−^CD4^−^CD8^+^ T cells among 7AAD^−^ LP cells from the indicated mice is shown. Data are pooled from four independent experiments that examined a total of 14–21 animals per group. **(C)** The percent of total B cells, identified as CD4^−^CD8^−^MHCII^+^B220^+^ cells, among 7AAD^−^ LP cells from the indicated mice is shown. **(D)** The number of IgA- or IgG-producing cells among CD11b^−^ LP lymphocytes determined by ELISPOT from the indicated mice is shown. **(E)** The percent of total T cells, identified as 7AAD^−^MHCII^−^CD3^+^ cells, from human colon LP is shown. Non-inflamed controls n = 10, UC patients in remission n = 6, UC patients with active inflammation n = 10. Data in C–D show the median of 15–24 mice per group examined in 4–6 independent experiments. Statistical significance was assessed using the Mann-Whitney- U-Test and Kruskal-Wallis test followed by Dunn’s multiple comparison test; significance is indicated as *p<0.05, **p<0.01, ***p<0.001, while all other comparisons are non-significant. For all panels, each symbol represents an individual mouse or patient. The mice used were between 7–19 weeks of age.
(TIF)

Figure S2 **Lack of correlation between age and inflammatory status in Muc2^−/−^ mice.** **A** PMN influx in colon LP in correlation to age is plotted. Pooled data from 14 experiments with 11–24 mice per group is depicted. **B** Differential gene expression in colon LP assessed by qPCR and determined using 2^ΔΔCT^ method with HPRT as the endogenous reference gene is plotted against the age of the corresponding mouse. Pooled data from 8 independent experiments with a total of 9–11 mice per group is shown. Analysis was performed using Pearson correlation. *p*-values and R^2^ values are indicated. The mice used were between 8–19 weeks of age.
(TIF)

Figure S3 **PMN influx and changes in myeloid cell populations are not apparent in the small intestine.** LP cells from the distal small intestine of Muc2^−/−^ mice, inflamed Muc2^−/−^ mice and Muc2^+/−^ controls were stained for DCs, PMN and macrophages and analyzed by flow cytometry as in Figs. 3 and 4. Data pooled from 5 independent experiments with a total of 5–9 mice per group is depicted. Each symbol represents an individual mouse. No statistical significant difference was detected using the Kruskal-Wallis test followed by Dunn’s multiple comparison test. Mice used were older than 11 weeks of age.
(TIF)
