# Supplementary figures and images for: Spontaneous Colitis in Muc2-Deficient Mice Reflects Clinical and Cellular Features of Active Ulcerative Colitis
Source: PLoS One. 2014 Jun 19;9(6):e100217. doi: 10.1371/journal.pone.0100217 (PMC4063762; doi:10.1371/journal.pone.0100217)

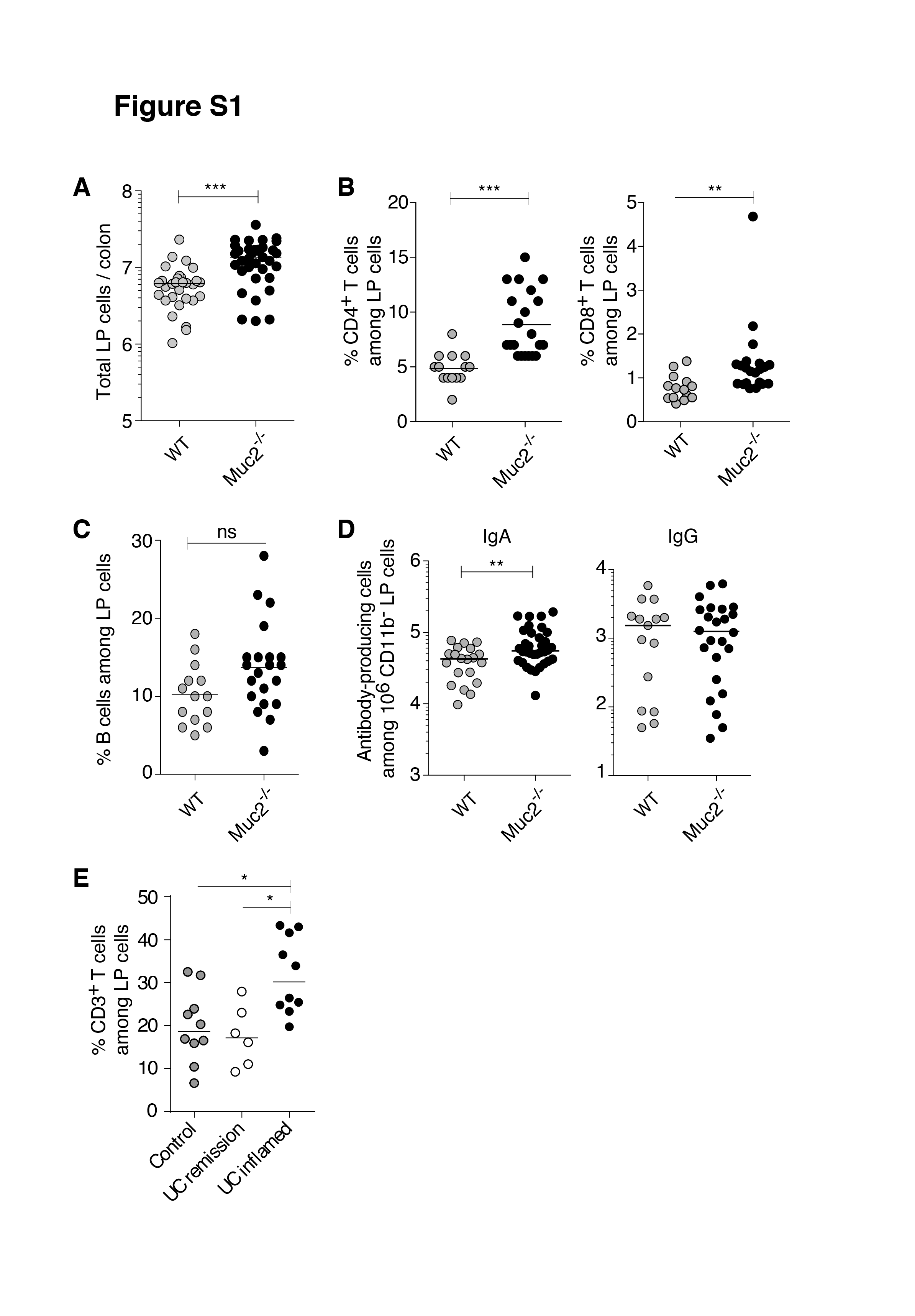

Supplement: Figure S1 — Lymphocytes increase in the colon LP of Muc2−/− mice and UC patients with active inflammation. LP cells were prepared from the colon of Muc2−/− mice and WT mice and from biopsies from UC patients with active inflammation, from UC patients in clinical remission and non-inflamed controls and were analyzed by flow cytometry. (A) The median number of total, viable (7AAD−) cells from colon LP from 21 WT and 35 Muc2−/− mice from 7 independent experiments is shown. (B) The percent B220−MHCII−CD8−CD4+ T cells or B220−MHC-II−CD4−CD8+ T cells among 7AAD− LP cells from the indicated mice is shown. Data are pooled from four independent experiments that examined a total of 14–21 animals per group. (C) The percent of total B cells, identified as CD4−CD8−MHCII+B220+ cells, among 7AAD− LP cells from the indicated mice is shown. (D) The number of IgA- or IgG-producing cells among CD11b− LP lymphocytes determined by ELISPOT from the indicated mice is shown. (E) The percent of total T cells, identified as 7AAD−MHCII−CD3+ cells, from human colon LP is shown. Non-inflamed controls n = 10, UC patients in remission n = 6, UC patients with active inflammation n = 10. Data in C–D show the median of 15–24 mice per group examined in 4–6 independent experiments. Statistical significance was assessed using the Mann-Whitney- U-Test and Kruskal-Wallis test followed by Dunn’s multiple comparison test; significance is indicated as *p<0.05, **p<0.01, ***p<0.001, while all other comparisons are non-significant. For all panels, each symbol represents an individual mouse or patient. The mice used were between 7–19 weeks of age. (TIF) [file pone.0100217.s001.tif]

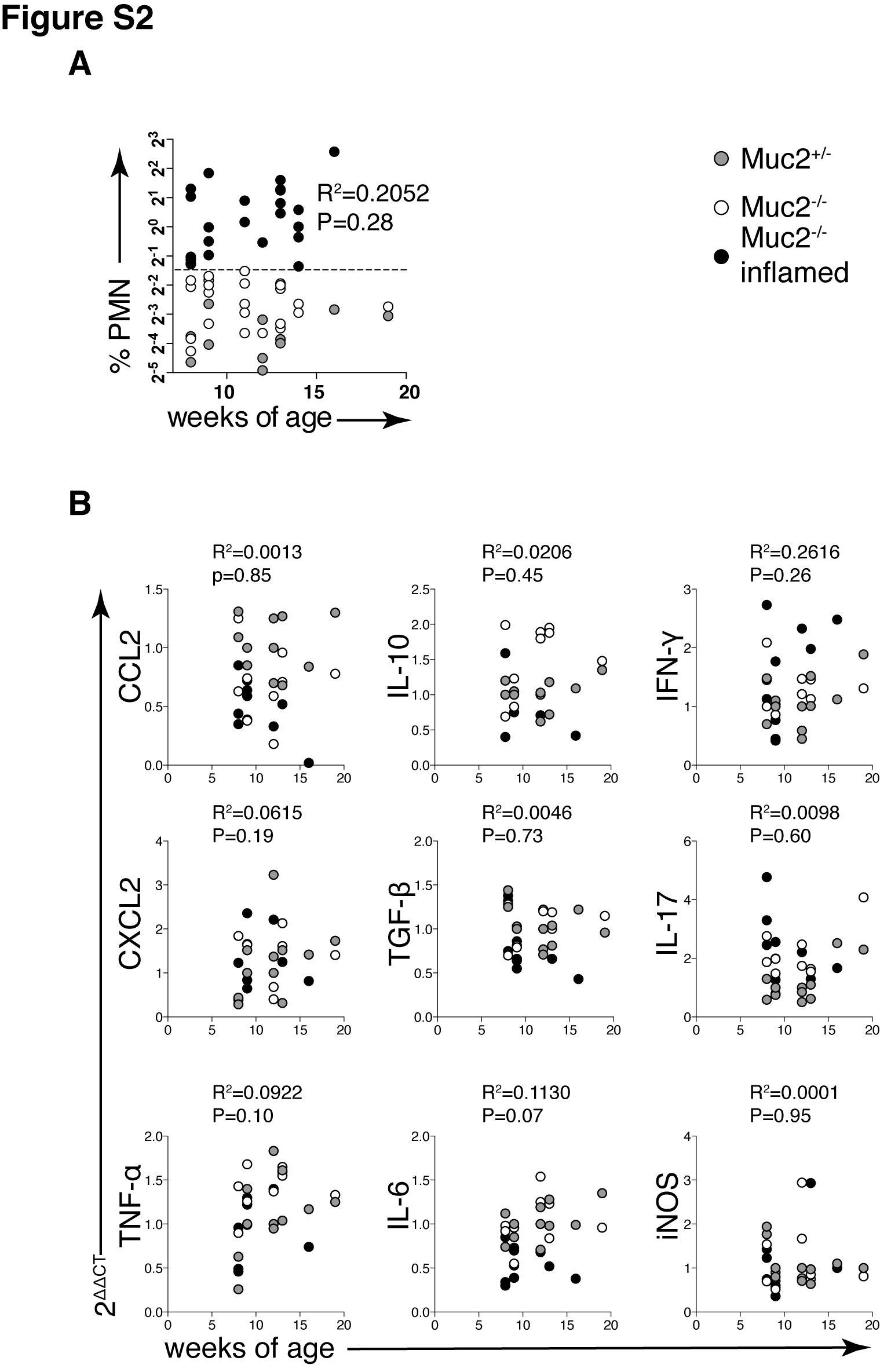

Supplement: Figure S2 — Lack of correlation between age and inflammatory status in Muc2−/− mice. A PMN influx in colon LP in correlation to age is plotted. Pooled data from 14 experiments with 11–24 mice per group is depicted. B Differential gene expression in colon LP assessed by qPCR and determined using 2ΔΔCT method with HPRT as the endogenous reference gene is plotted against the age of the corresponding mouse. Pooled data from 8 independent experiments with a total of 9–11 mice per group is shown. Analysis was performed using Pearson correlation. p-values and R2 values are indicated. The mice used were between 8–19 weeks of age. (TIF) [file pone.0100217.s002.tif]

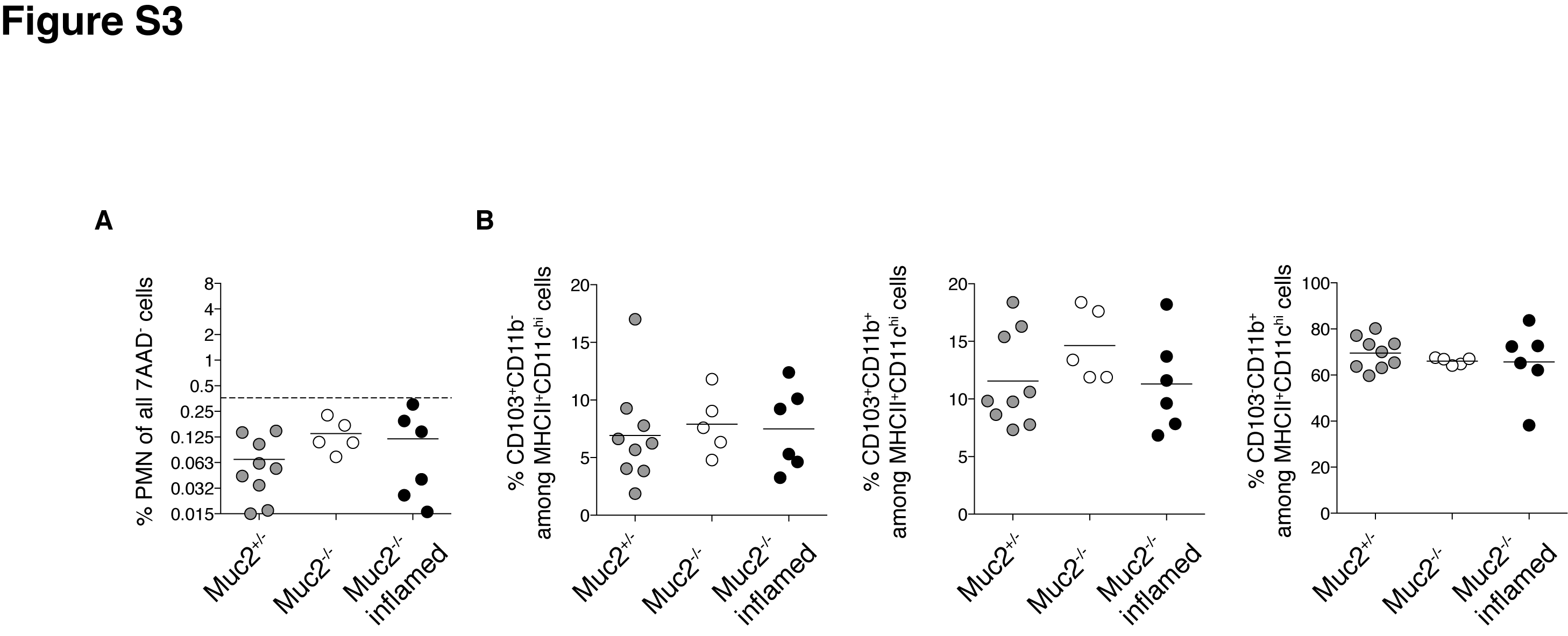

Supplement: Figure S3 — PMN influx and changes in myeloid cell populations are not apparent in the small intestine. LP cells from the distal small intestine of Muc2−/− mice, inflamed Muc2−/− mice and Muc2+/− controls were stained for DCs, PMN and macrophages and analyzed by flow cytometry as in Figs. 3 and 4. Data pooled from 5 independent experiments with a total of 5–9 mice per group is depicted. Each symbol represents an individual mouse. No statistical significant difference was detected using the Kruskal-Wallis test followed by Dunn’s multiple comparison test. Mice used were older than 11 weeks of age. (TIF) [file pone.0100217.s003.tif]
